# Twenty-four-month interim results from a prospective, single-arm clinical trial evaluating the performance and safety of cellular bone allograft in patients undergoing lumbar spinal fusion

**DOI:** 10.1186/s12891-023-06996-5

**Published:** 2023-11-17

**Authors:** Daniel K. Park, Joshua J. Wind, Todd Lansford, Pierce Nunley, Timothy A. Peppers, Anthony Russo, Hamid Hassanzadeh, Jonathan Sembrano, Jung Yoo, Jonathan Sales

**Affiliations:** 1 Beaumont Hospital, 3601 W 13 Mile Rd., Royal Oak, MI USA; 2 Sibley Memorial Hospital, 5255 Loughboro Rd. NW, Washington DC, USA; 3 South Carolina Sports Medicine, 9100 Medcom, N Charleston, SC USA; 4 Spine Institute of Louisiana, 1500 Line Ave, Shreveport, LA USA; 5 Scripps Memorial Hospital Encinitas, 354 Santa Fe Drive, Encinitas, CA USA; 6 Yellowstone Orthopedic and Spine Institute, Billings Clinic Bozeman , 3905 Wellness Way, Bozeman, MT , MT USA; 7 John Hopkins Medicine, 6420 Rockledge Dr, Bethesda, MD USA; 8 University of Minnesota, 909 Fulton St SE, Minneapolis, MN USA; 9 Oregon Health and Science University, 3181 SW Sam Jackson Park Rd, Portland, OR USA; 10 Summit Spine, 9155 SW Barnes Rd. 210, Portland, OR USA

**Keywords:** Lumbar fusion, Arthrodesis, Cellular allograft, Trinity Elite, CBA

## Abstract

**Background:**

Autologous bone grafts are the gold standard for spinal fusion; however, harvesting autologous bone can result in donor site infection, hematomas, increased operative time, and prolonged pain. Cellular bone allografts (CBAs) are a viable alternative that avoids the need for bone harvesting and may increase fusion success alone or when used as an adjunct material. The present study examined the efficacy and safety of CBA when used as an adjunct graft material to lumbar arthrodesis.

**Methods:**

A prospective, single-arm, multicenter clinical trial (NCT 02969616) was conducted in adult subjects (> 18 years of age) undergoing lumbar spinal fusion with CBA graft (CBA used as primary (≥ 50% by volume), with augmentation up to 50%). Radiographic fusion status was assessed by an independent review of dynamic radiographs and CT scans. Clinical outcomes were assessed with the Oswestry Disability Index (ODI), and Visual Analog Scales (VAS) score for back and leg pain. Adverse events were assessed through the 24-month follow-up period. The presented data represents an analysis of available subjects (n = 86) who completed 24 months of postoperative follow-up at the time the data was locked for analysis.

**Results:**

Postoperative 24-month fusion success was achieved in 95.3% of subjects (n = 82/86) undergoing lumbar spinal surgery. Clinical outcomes showed statistically significant improvements in ODI (46.3% improvement), VAS-Back pain (75.5% improvement), and VAS-Leg pain (85.5% improvement) (p < 0.01) scores at Month 24. No subject characteristics or surgical factors were associated with pseudoarthrosis. A favorable safety profile with a limited number of adverse events was observed.

**Conclusions:**

The use of CBA as an adjunct graft material showed high rates of successful lumbar arthrodesis and significant improvements in pain and disability scores. CBA provides an alternative to autograft with comparable fusion success rates and clinical benefits.

**Trial registration:**

NCT 02969616.

## Background

Lumbar fusion is frequently used for a variety of degenerative, traumatic, and oncologic conditions of the lumbar spine. Advancements in surgical techniques, including spinal instrumentation and combined anterior and posterior procedures have led to increased rates of fusion. Autologous iliac crest bone graft (ICBG) is the gold standard bone graft and offers osteoconductive, osteoinductive and osteogenic properties that aid in achieving successful fusion. However, harvesting ICBG may result in donor site infection, hematomas, increased operative time, prolonged pain, altered cosmesis, and in rare instances arterial and nerve injury [1–3]. Alternatives to autograft include bone marrow aspirate, bone graft allografts, synthetic bone void fillers, and bone morphogenetic protein [4, 5]. Although these alternative bone graft options provide one or two healing properties, they lack the complete trio of osteoconductive, osteoinductive and osteogenic properties found in autograft bone.

Cellular bone allografts (CBAs) provide an innovative addition to allograft technologies and are an alternative to other bone grafting options. From a structural standpoint, allograft may serve in the absence of revascularization and creeping substitution processes. Cellular bone allografts provide the same three osteometric properties promoted with ICBG without the complications of donor site morbidity associated with bone graft harvesting, which has been reported to be as high as 38% [6–11]. Although CBAs have been utilized in lumbar and cervical spinal fusion surgery, there is limited clinical literature detailing fusion rates and the impact of selected patient demographics and associated outcomes on clinical measures [12–16].

A prospective, post marketing, multicenter, open label, non-randomized clinical study was conducted to assess the efficacy and safety of CBA (Trinity Elite^®^) in lumbar fusion surgery. The current report presents an interim analysis of the clinical and radiographic outcomes at 24-months of postoperative follow-up.

## Methods

As previously published and described in the 12-month interim analysis by Wind et al. [17], this prospective, single-arm, open label study enrolled subjects prospectively at nine clinical sites and screened for enrollment via preset inclusion/exclusion criteria. All participating sites received IRB approval prior to initiation of the study. Sites used a central IRB (Western IRB or WCG IRB) or local IRB (University of Virginia and the Oregon Health & Science University). Subjects older than 18 years of age, that had failed at least six months of conservative care, who planned to undergo posterolateral fusion (1–4 levels) or interbody fusion (1–2 levels), and met the predefined inclusion/exclusion criteria were enrolled. Subjects who underwent a prior lumbar spinal fusion surgery at a level currently scheduled for surgery, were undergoing treatment for malignancy, or had undergone treatment for malignancy within the last five years (benign skin cancer permitted), presented with an active local or systemic infection, or were undergoing adjunctive treatment for local or systemic infections were excluded. The surgical approach, technique, and placement/location of the bone graft was determined at the discretion of the treating surgeon. Subjects received CBA (Trinity Elite®, MTF Biologics, Edison NJ) as the primary bone graft substance (≥ 50% by volume), with augmentation of up to 50% of locally harvested autograft and/or cancellous allograft chips. No additional bone graft substitutes were allowed. No tissue was harvested from the iliac crest. Subject demographics and baseline characteristics including risk factors for pseudoarthrosis were recorded.

Dynamic x-rays (flexion/extension) were obtained at 3 months, 6 months, 12 months, and 24 months postoperatively, while computerized tomography (CT) scans were obtained at 12 and 24 months. Radiographic fusion at 24 months was assessed by an independent reviewer (TELOS Partners, Warsaw, IN, and MMI, Houston TX), with successful fusion being defined as: (1) lack of angular and translational motion (< 3° and < 3 mm, respectively) on Quantitative Motion Analysis (QMA) and (2) the presence of bridging bone across the adjacent endplates, or transverse processes on thin-cut CT scans. Both fusion criteria had to be met for the subject to be considered a fusion success. In multi-level procedures, all treated levels had to be fused to be considered a fusion success. Clinical outcomes included the Oswestry Disability Index (ODI), and Visual Analog Scales (VAS) for back and leg pain. Clinical outcomes were obtained preoperatively and 6 weeks, 3 months, 6 months, 12 months, and 24 months postoperatively. Adverse events were recorded from surgery through 24 months for each subject, including the event’s relatedness, severity, and outcome.

Subject data was analyzed with SAS Version 9.4 (SAS Institute, Cary, NC). Counts and percentages were reported for categorical baseline variables; the mean standard deviation (SD) and range were reported for continuous variables. Preoperative and postoperative subject reported outcomes were compared with a Wilcoxon signed rank test. Correlation of outcomes to risk factors were calculated by chi-square test. Alpha was set at 0.05 and a p-value of ≤ 0.05 was considered significant.

## Results

### Subject demographics and surgical procedure

At the time of this interim analysis, 86 subjects had completed 24-months postoperative radiographic and clinical evaluations. The mean age was 59.2 ± 11.9 years (range 28–82) and the subject population included a higher number of females (n = 57, 66.3%) to males (n = 29, 33.7%) with a mean body mass index (BMI) of 30.1 ± 6.3 kg/m^2^ (range 18.9–44.6). Twelve subjects were current smokers (14.0%), 17 were diabetic (19.8%) and 8 presented with osteoporosis (9.3%). Twenty-two subjects (25.6%) underwent multi-level lumbar arthrodesis. Subject demographics are presented in Table 1.

**Table 1:** Subject Demographics

Subject Demographic	Subjects n = 86
Gender, n (%) Female Male	57 (66.3) 29 (33.7)
Age, years Mean (SD)	59.2 (11.9)
BMI (kg/m^2^) Mean (SD)	30.1 ± 6.3
Nicotine User, n (%)	12 (14.0)
Diabetics, n (%)	17 (19.8)
Multi-Level Disease, n (%)	22 (25.6)
Osteoporosis, n (%)	8 (9.3)
Use of Autograft/Allograft	38 (44.2)

### Radiographic fusion outcomes

**Fig. 1:**
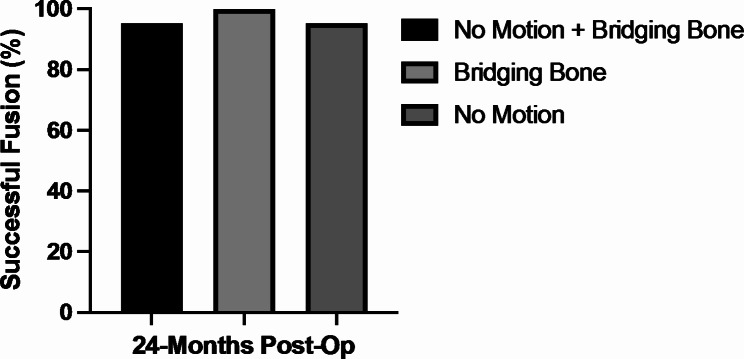
Fusion Success at 24 Months. Subjects showed high rates of fusion success across all groups with a range from 95.3 − 100.0%.

**Fig. 2:**
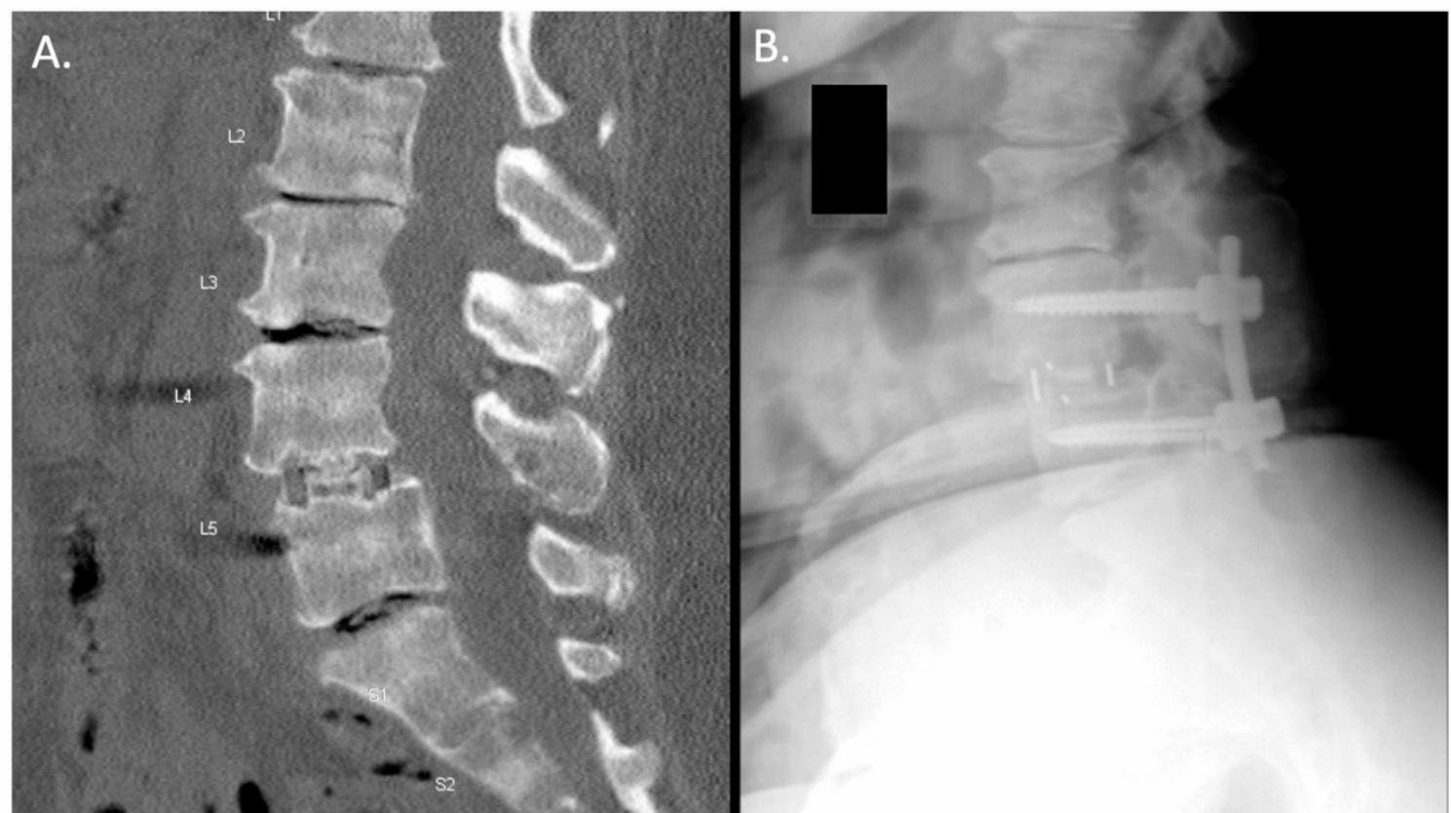
Spinal Fusion Success. 24-Month postoperative imaging demonstrating successful lumbar fusion. *L, lumbar vertebrae*

Fusion success was confirmed in 95.3% of subjects (n = 82/86). There were 4 subjects (4.7%) with evidence of pseudoarthrosis, with ≥ 3° of angular motion on QMA, although bridging bone was present. No revisions were reported for these 4 subjects at the time of this report (Fig. 1). A representative image depicting successful fusion at 24-months is presented (Fig. 2).

### Fusion success by levels treated

There were 60 subjects (69.8%) that underwent a 1-level procedure, 21 subjects (24.4%) underwent a 2-level procedure, and 1 subject (1.2%) underwent a 4-level procedure. Fusion rates for single and multi-level disease were 93.7% and 100.0%, respectively (Table 2).

### Fusion success by surgical approach

Nineteen subjects (22.1%) underwent anterior lumbar interbody fusion (ALIF), 21 subjects (24.4%) underwent lateral or oblique lateral lumbar interbody fusion (LLIF, DLIF, XLIF or OLIF), 35 subjects (40.7%) underwent a posterior interbody fusion (PLIF, MIDLIF or TLIF), and 11 subjects (12.8%) underwent posterolateral lumbar fusion (PLF). The rate of fusion success was high regardless of the surgical approach (Table 2).

**Table 2:** Fusion Success by Level and Surgical Approach.

Surgical Parameter	Fusion Success n (%)
Levels Treated	
1 level	60/64 (93.7)
2 levels	21/21 (100.0)
3 levels	0/0 (0.0)
4 levels	1/1 (100.0)
Surgical Approach	
ALIF	17/19 (89.5)
Lateral or Oblique Lateral [OLIF, XLIF, LLIF, DLIF]	21/21 (100.0)
Posterior Interbody [TLIF, MIS-TLIF, PLIF]	33/35 (94.3)
PLF	11/11 (100.0)

*ALIF, anterior lumbar interbody fusion; DLIF, direct lateral lumbar interbody fusion; LLIF, lateral lumbar interbody fusion; MIS-TLIF, minimally invasive transforaminal lumbar interbody fusion; OLIF, oblique lateral interbody fusion; PLIF, posterior lumbar interbody fusion; PLF, posterior lumbar fusion; TLIF, transforaminal lumbar interbody fusion; XLIF, extreme lateral interbody fusion.*

### Clinical outcomes

**Fig. 3:**
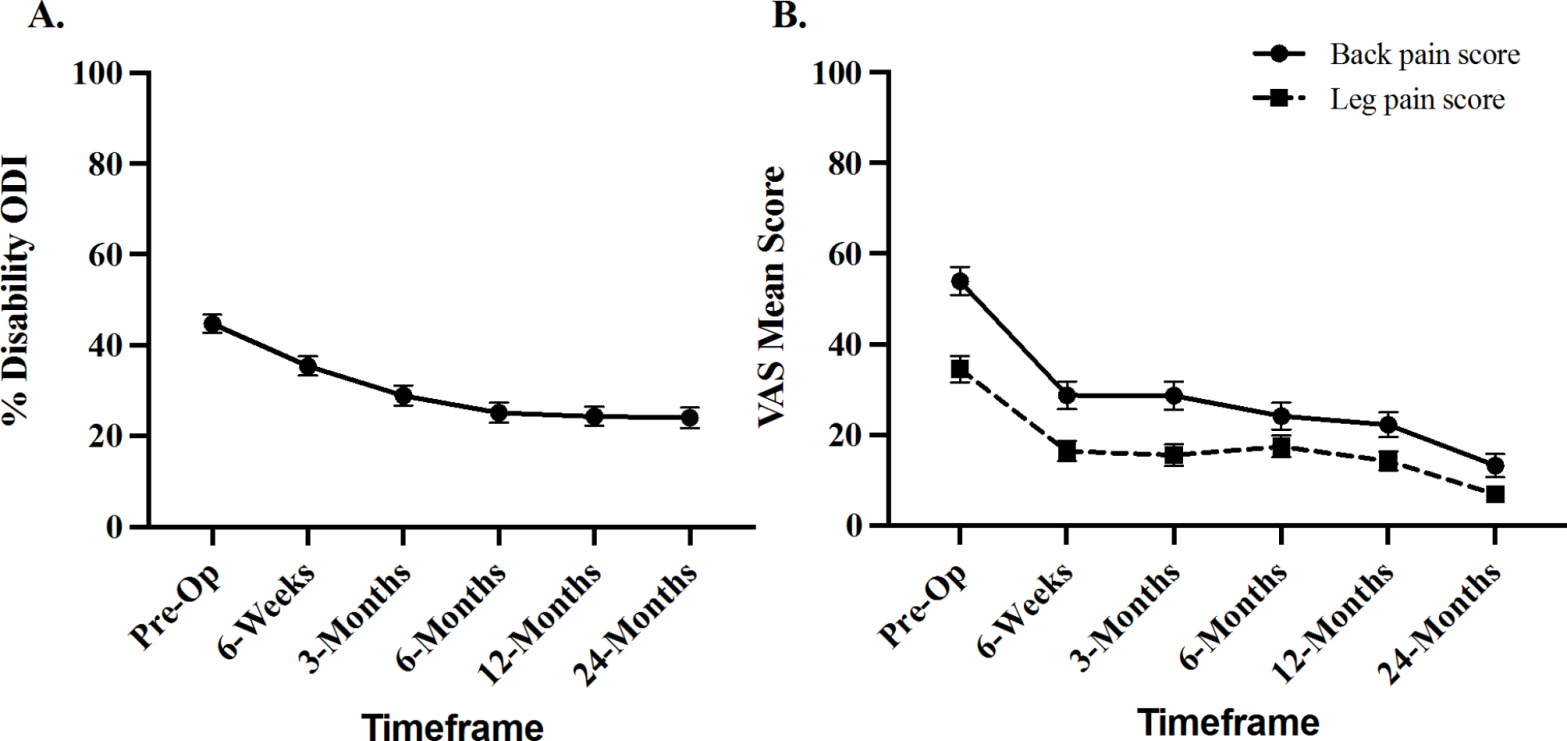
ODI Percentage of Disability and VAS Pain Scores Following Lumbar Spinal Surgery. (**A**) ODI Percentage of Disability and (**B**) VAS (Total back and leg) pain scores improved following surgery through the 24-month postoperative visit.

The percentage of disability as assessed by the ODI decreased from 44.7% ± 18.4 preoperatively, to 24.0% ± 21.2 (p < 0.001) at 24 months post-op (Fig. 3A). In subjects that did not have successful fusion (n = 4), the preoperative mean disability was 37.0% ± 7.4 and improved to 6.5% ± 11.7 (p = 0.13). An overall improvement of 75.5% was observed in VAS-Back scores at 24 months post-op. The preoperative mean VAS-Back pain score was 53.9 ± 28.6 and improved to 13.2 ± 23.3 (p < 0.0001) at 24 months post-op. In subjects that did not have successful fusion (n = 4), the preoperative mean VAS-Back pain score was 55.3 ± 29.8 and improved to 8.0 ± 14.1 (p = 0.03). An overall improvement of 85.5% was observed in VAS-Leg pain scores at 24 months post-op. The preoperative mean VAS-Leg pain score was 34.5 ± 25.9 and improved to 6.9 ± 15.6 (p < 0.0001) at 24 months post-op(Fig. 3B). In subjects that did not have successful fusion (n = 4), the preoperative mean VAS-Leg pain score was 26.4 ± 13.3 and improved to 1.1 ± 1.3 (p < 0.001).

Subjects were further evaluated for fusion success by risk factor. No risk factors significantly impacted fusion success (Table 3).

**Table 3:** Fusion Success Rates by Risk Factor.

Risk Factor	Fusion Success n (%)
BMI ≥ 30	40/40 (100.0)
Smoking	12/12 (100.0)
Age (65 + years)	28/29 (96.5)
Diabetes	17/17 (100.0)
Osteoporosis	8/8 (100.0)
Multi-level disease	22/22 (100.0
Multiple risk factors	38/38 (100.0)

*BMI, body mass index.*

### Adverse events

Adverse events (AEs) were reported and characterized by relatedness and severity. Three AEs were considered related to the graft/surgical procedure. A single serious AE (postoperative radiculopathy) was reported as definitely related to the bone graft. Surgical exploration revealed extrusion of the graft material from the interbody. This subject demonstrated successful fusion at 12 months. Two additional AEs reported as probably related to the surgical procedure included hyposthesia and increased lower back pain. Hypoesthesia, which resulted in numbness/loss of sensation in the left lower extremity, was categorized as non-serious and treated with unspecified medication. Increased lower-back pain resulted in the subject undergoing a two-level laminectomy and fusion. Demographics, baseline characteristics, and risk factors for these three subjects are presented in Table 4.

**Table 4:** Demographics and Baseline Variables for Subjects with Adverse Events Related to the Graft or Surgical Procedure.

	Subject 1 (SAE) Extruded Graft	Subject 2 (SAE) Lami/Fusion	Subject 3 (AE) Hypoesthesia	
Age	69	31		48
Sex	Female	Female		Male
Race	Caucasian	Caucasian		Caucasian
Height (in)	66	62		70
Weight (lb)	178	185		258
BMI	28.7	33.8		37.0
Diabetes	No	No		No
Current Nicotine Use	No	Yes		Yes
Osteoporosis	Yes	No		No

## Discussion

Despite the dramatically higher number of lumbar spinal fusion procedures over the past decade, the potential for pseudoarthrosis persists [18, 19]. Surgical treatment using autologous ICBG is considered the gold standard for spinal fusion [20–23]. However, ICBG is associated with complications, such as donor site pain, infection, and prolonged recovery. While local bone at the surgical site may be available, the supply is limited; thus, the need for alternative bone grafting that can be used as a stand-alone graft or as an adjunct is of high interest [1, 2]. The current clinical trial evaluated CBA as an adjunct bone graft to autograft and demonstrated a high rate of fusion with improved clinical outcomes and a favorable safety profile.

Cellular bone allografts are an alternative to autograft given they have all three principal components of a bone graft, namely osteoconductivity, osteoinductivity, and osteogenicity. Several studies provide support for high fusion rates using CBA in keeping with the current report. Musante et al. report 90.0% fusion success in procedures using viable osteogenic cells, with no differences in reported fusion rates in patients with and without risk factors [24]. Other CBAs, including Vivigen (DepuySynthes, Raynham MA), have shown spinal fusion rates at 90.0% [25]. In a study of multi-level fusion in a posterolateral construct with CBA, a fusion rate of 98.7% was observed [15]. Elgafy et al., report a fusion rate of 91.7% with CBA in 96 patients and 222 treated levels [13]. Furthermore, Ammerman et al. report 91.3% successful fusion in minimally invasive TLIF cases using CBA in 23 patients totaling 26 levels [15]. In extreme lateral interbody (XLIF) procedures, Tohmeh et al. report a fusion rate of 90.2% in 40 patients with a total of 61 levels [16]. In comparison, fusion success rates with the gold standard autograft have been reported in a range from 65.0 to 93.0% [13, 20–23]. Altogether, the current report further supports data showing high fusion rates using CBA, comparable and even superior to those obtained using autograft.

Significant improvements in clinical outcomes evaluating patient-reported levels of disability and pain were observed. All clinical outcome measures met statistical and clinical significance criterion, further demonstrating the positive impact and meaningful improvement from lumbar spinal fusion in this population. These findings are consistent with other reports showing significant improvements in ODI and VAS disability and pain scores following surgical procedures with CBA. Tohmeh et al. report a 41.0%, 55.0% and 43.0% improvement in ODI, VAS-Back and VAS-leg pain scores postoperatively at 12 months, respectively [16] [13]. Our findings show greater improvements in these outcomes positing a benefit to the specific bone graft used (Trinity allograft) and the adjunctive use with autograft. Further investigation is necessary to determine optimized procedures that provide maximal impact on these patient-reported outcomes.

Of note, the small number of subjects that did not have successful fusion also showed improved clinical outcomes. If a solid fusion is not obtained, but stability to the spine is supported through the instrumentation, the patient may still achieve clinical improvements. Lack of angular and translational motion in addition to bridging bone was the per-protocol definition of fusion. For the majority of subjects with a failed fusion, bridging bone was present which likely contributed to the stability of the spine even though they failed the QMA assessment.

Fusion success and clinical outcomes may be affected by patient risk factors for pseudoarthrosis, including older age, osteoporosis, alcoholism, malnutrition, and smoking [26–28]. In this study, subset analysis of subjects with known risk factors for pseudoarthrosis found that fusion rates were not significantly different between subjects with a single risk factor or multiple risk factors. These findings corroborate other reports of successful fusion and improvements in patient reported outcomes, despite comorbidities and risk factors that are known to negatively impact these outcomes [13].

The study presented several limitations that should be noted. Varying surgical approaches (e.g., TLIF, ALIF, PLF) were included within this analysis, as opposed to a single approach. This analysis does not include a control arm for comparison, the only comparison to outcomes identified within come from the available scientific literature. Despite these limitations, the preliminary data provided within this report demonstrates that CBA is an effective bone graft alternative and adjunct for patients being treated with lumbar spinal fusion.

Allograft infection can lead to disastrous complications such as nonunion of the graft-host junction and fracture of the graft requiring surgical intervention. While there has been a recent report of disease transmission through the usage of a different CBA where multiple tuberculosis cases in graft recipients were traced to a single donor lot [29], allogeneic bone has an extensive clinical safety record and Trinity allografts have never been linked to donor-derived infections after more than 13 years of being commercially available [30–32]. Prior to this incident, only one other occurrence of suspected tuberculosis disease transmission through the transplantation of allograft bone was identified in the published literature and that was reported nearly 70 years ago before modern donor screening criteria were established [33]. In order to address the risk of disease transmission with Trinity allografts, the medical and social history of each donor is carefully screened prior to donation for medical conditions or disease processes that would contraindicate the donation of tissue in accordance with current policies and criteria that have been established by FDA and the American Association of Tissue Banks (AATB). Subsequently, the processing and packaging of Trinity Elite allografts are performed under controlled aseptic conditions in an ISO Class 4 environment. Following processing, all donor batches are evaluated for sterility and must show no evidence of microbial growth complying with USP < 71 > Sterility Testing. Donor consent, infectious disease test results, medical history interviews, available medical records and any other information that may pertain to donor eligibility are evaluated by a medical director and must be deemed to be sufficient to indicate that donor tissues are suitable for transplantation [34, 35].

## Conclusions

The use of CBA as a graft or primary adjunct material resulted in fusion rates comparable to ICBG and consistent across the entire patient population. Successful fusion was attained regardless of risk factor reported, and without the drawback of donor site morbidity and complications associated with bone morphogenetic protein products. This study adds significant value to current literature regarding CBAs and their efficacy in spinal fusion and provides an update to previously published data from the 12-month interim analysis of the study [17]. By preserving the inherent properties of these grafts, including the osteoinductive and osteogenic components retained within the bone matrix, CBAs provide a unique alternative and adjunct graft material to autograft.

## Data Availability

The datasets used and/or analyzed during the current study are available from the corresponding author on reasonable request.
